# Current Knowledge on Pathogenicity and Management of *Stemphylium botryosum* in Lentils (*Lens culinaris* ssp. *culinaris* Medik)

**DOI:** 10.3390/pathogens8040225

**Published:** 2019-11-08

**Authors:** Arpita Das, Subrata Dutta, Subhendu Jash, Ashis Roy Barman, Raju Das, Shiv Kumar, Sanjeev Gupta

**Affiliations:** 1 Bidhan Chandra Krishi Viswavidyalaya, Mohanpur, Nadia, West Bengal 741252, India; arpitacoh@gmail.com (A.D.); subratadutta1972@gmail.com (S.D.); drsubhendujash@gmail.com (S.J.); ashisroybarman@gmail.com (A.R.B.); rajudas05@gmail.com (R.D.); 2 International Centre for Agricultural Research in the Dry Areas (ICARDA), Rabat- Institutes, B.P. 6299 Rabat, Morocco; 3 All India Coordinated Research Project (AICRP) on MULLaRP, ICAR- Indian Institute of Pulses Research, Kanpur, Uttar Pradesh 208024, India

**Keywords:** epidemiology, integrated disease management, lentil, *Stemphylium botryosum*

## Abstract

Stemphylium blight (SB) caused by Ascomycete, *Stemphylium botryosum* Wallr. has been a serious threat to lentil cultivation, mainly in Bangladesh, Nepal, India, and Canada since its first outbreak in Bangladesh in 1986. The genus *Stemphylium* Wallr., a dematiaceous hyphomycete, comprises up to 150 species, and is pathogenic on a wide range of plants infecting leguminous as well as nonleguminous crops. In recent years, studies indicated overlapping in morphological characters among the different species under the genus *Stemphylium*, making the identification and description of species difficult. This necessitates different molecular phylogenetic analysis in species delimitation. Therefore, a detailed understanding of spatial diversity and population structure of the pathogen is pertinent for producing source material for resistance breeding. The role of different weather variables as predisposing factors for the rapid spread of the pathogen necessitates devising a disease predictive model for the judicial application of fungicides. A dearth of information regarding spore biology, epidemiology, race diversity, host-pathogen interaction, and holistic disease management approach necessitates immediate attention towards more intensive research efforts. This is the first comprehensive review on the current state of knowledge and research efforts being made for a better understanding of the SB resistance through cognizing biology, ecology, and epidemiology of *S*. *botryosum* and effective disease management strategies to prevent widespread outbreaks of SB. The information regarding the biology and epidemiology of *S*. *botryosum* is also crucial for strengthening the “Integrated Disease Management” (IDM) programme. The need for a regional research network is advocated where the disease is becoming endemic.

## 1. Introduction

Lentil (*Lens culinaris* ssp. *culinaris* Medik.) is the world’s fifth largest pulse crop cultivated in more than 70 countries around the world, mainly in West Asia, North Africa, the Indian subcontinent, North America, and Australia [1]. Based on nutritional properties, it has been recognized as one of the five healthiest foods [2] as lentil grains are high in protein, fiber, micronutrients, and vitamins [3,4].

Stemphylium blight (SB) caused by the Ascomycete, *Stemphylium botryosum* Walr, once a minor disease with local significance in South Asia, is now a serious threat to lentil cultivation in many parts of the world [5]. It is classified as a fungal disease responsible for large scale defoliation of plants, causing severe yield losses within a short period in conducive environments. It has been reported in lentil producing areas of Bangladesh, India, Nepal, the USA, and Canada [6,7]. The first appearance of SB was observed in Bangladesh in 1986 [8], where with increased severity it caused up to 80% yield losses [9,10]. The disease has been on the rise in frequency and intensity in India, which ranks first in lentil coverage areas globally. The disease holds the potential for causing much greater losses once it spreads to other lentil producing areas with favorable environmental conditions for the pathogen. The potential threat of its global spread warrants immediate attention to understand and manage this disease by developing a regional network where the disease is endemic. 

SB can easily be distinguished from other blights of lentil like Alternaria blight and Ascochyta blight based on symptoms, the severity of infection, and morphology of pathogen. *Alternaria* is closer to *Stemphylium*, and often mixed infection of both is encountered on lentils. Like SB, the Ascochyta blight and Alternaria blight generally appear in the field during the flowering stage in a humid, cooler climate in the presence of prolonged leaf wetness. The initial symptom of Ascochyta Blight is characterized by the formation of whitish to grayish lesions on the stems and leaves that turn light tan in color, and the mature lesions have darker margins with prominent black pycnidia scattered throughout the lesions. On the contrary, no dark pycnidial structure is found in lesions caused by SB.SB produces more pronounced symptoms on leaves. 

**Figure 1:**
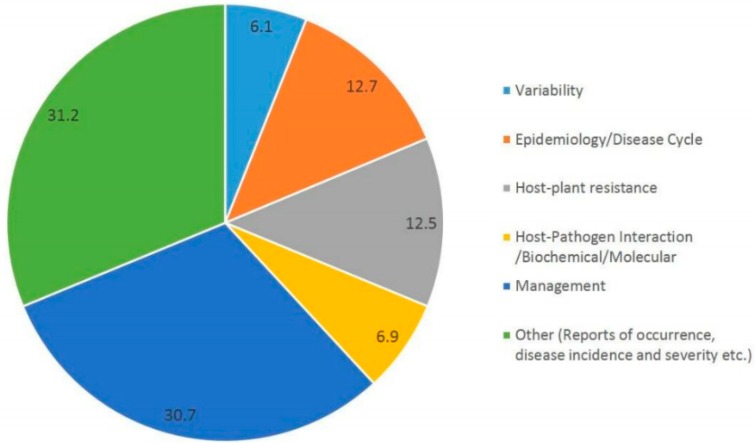
Overview of global research concerning various aspects of *Stemphylium* as incitant of Stemphylium blight (SB) disease of different host plants as published and indexed in Web of Science (WoS) for the period 1998–2019. The numbers in the chart represent percentage of research executed in the respective domain.

To date, the disease is poorly understood and very few studies were made on the epidemiology of the fungus, the factors affecting disease development, the racial structure of the population, the interactions with different hosts, and genetics of resistance. An overview of global research concerning various aspects of *Stemphylium* as incitant of SB disease of different host plants published and indexed in Web of Science (WoS) for the period of 1998–2019 indicated that nearly 770 articles were published on *Stemphylium* on different hosts, of which only 200 papers were published on *Stemphylium botryosum*. However, lentil *Stemphylium* covers only 4.6% of total global research, whereas, the pear *Stemphylium* pathosystem shares 12.6%, onion *Stemphylium* 10.4%, tomato *Stemphylium* 8.6%, and garlic *Stemphylium* covers 4.8% of global research. Moreover, it was found that nearly 43% of the research papers were on the disease management approach and little has been done on pathogenicity and breeding for resistance against this disease (Figure 1). Therefore, this article reviews the current knowledge about the history, etiology, epidemiology, variability, and host plant resistance, and discusses the future need for integrated disease management strategies. 

## 2. Emergence and Spread of SB

**Figure 2:**
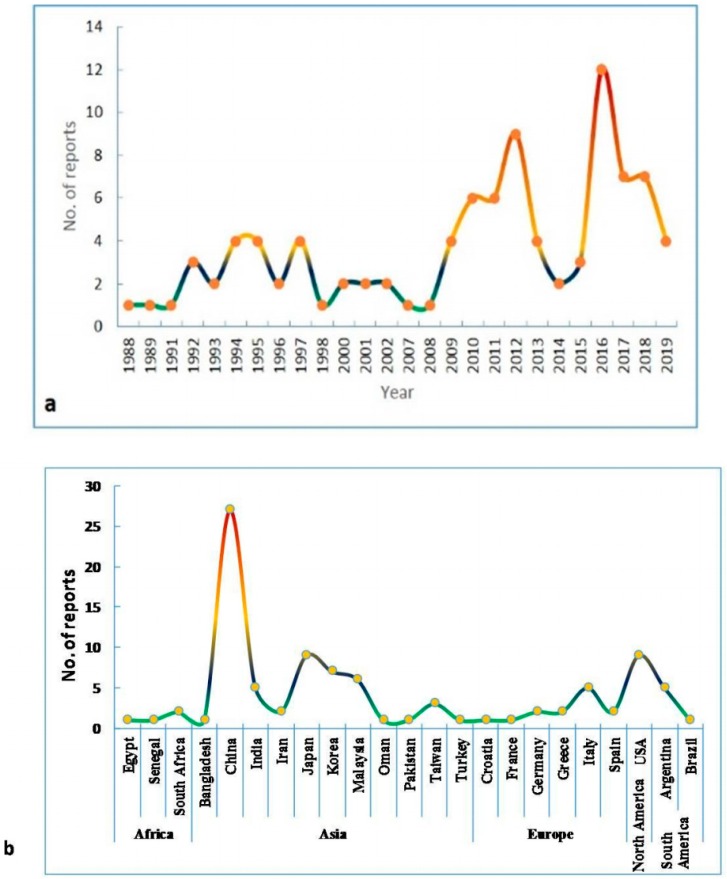
Reports of the first occurrence of various species of *Stemphylium* on different hosts. A total of 95 reports of *Stemphylium* spp. has been documented on various host plants in different countries from 1988 to 2019. (**a**). Year-wise number of reports published during1988 to 2019. (**b**). Number of reports of occurrence of SB disease in different countries of each continents.

SB in lentil, first reported in 1986 from Bangladesh, has later been observed in Hungary [11], India [12], Nepal [13], Canada [5], and Australia [14]. Subsequently, yield losses due to this disease were reported from many other countries of South Asia, Africa, and North America [6]. Among South Asian countries, Bangladesh and India are severely affected due to this dreaded disease [10]. In India, disease severity was observed up to 83% causing nearly 93% yield loss [15]. SB has been reported as a potential threat to lentil production in Western Canada [6]. There are increasingly more reports of pathogenic *Stemphylium* spp. in different countries on existing and new hosts (Figure 2). Congenial weather for SB coupled with the absence of resistant varieties may prompt serious outbreaks of the disease with the potential to cause huge yield losses in endemic areas. 

## 3. The Pathogen

*Stemphylium* is a species-rich genus which is pathogenic on more than 43 plant genera throughout the world and causes varying degrees of losses on different crops [16]. The genus *Stemphylium* Wallr. was first established in 1833 and comprises nearly 150 species [17]. Many of them are endophytic, epiphytic, or saprophytic in nature [18]. *S. botryosum* type species infects a range of crop plants in varying climatic conditions. Other host plants for *S. botryosum* are spinach [19,20], soybean, bean, pea, coriander, caraway and fenugreek, tomato, onion, clover and alfalfa [21,22], common bean, faba bean, etc. Legumes are generally infected either by *S. sarciniforme* or *S. Botryosum* [23]. 

**Figure 3:**
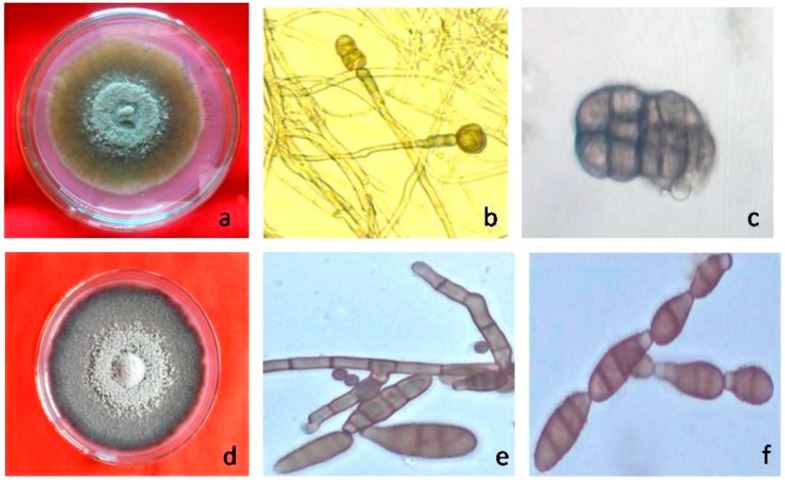
Colony morphology, conidiophore and conidia of *Stemphylium* spp. and *Alternaria* spp. (**a**). Colony morphology of *Stemphylium* spp. (**b**). Conidiophore of *Stemphylium* spp. (**c**). Conidia of *Stemphylium* spp. (**d**). Colony morphology of *Alternaria* spp. (**e**). Conidiophore of *Alternaria* spp. (**f**). Conidia of *Alternaria* spp.

Morphologically, the genus *Stemphylium* can be distinguished from other related genera like *Alternaria* and *Ulocaladium* with proliferating conidiophores and apically swollen conidiogenous cells. The shape and size of conidia, conidiophores, and ascospores are useful for species identification [24]. Recent studies to differentiate species in the genus *Stemphylium* have demonstrated overlapping in morphological characters, making the identification and description of species difficult. Based on previous study, different *Stemphylium* isolates have been placed into three different morphological groups based on colony character and conidial morphology [17]. *S*. *callistephi*, *S*. *lycopersici* and *S*. *solani* are similar in conidial shape and size, but other characteristics make them distinct. Based on conidial size alone, *S. trifolii* is like *S*. *eturmiunum*, but *S*. *trifolii* has smooth, pointy, regular dictyoconidia that are pale in color, with one dark transverse septum and no prominent constriction. Likewise, *S. majusculum* has conidia appearing similar to *S*. *vesicarium*, but their larger size and slightly more rectangular shape make them distinguishable (Table 1). Among these five closely related genera only *Alternaria* and *Stemphylium* are pathogenic to lentil, therefore these need to be identified based on colony culture and conidial morphology (Figure 3). 

Little work has been made to differentiate species in the genus *Stemphylium* at molecular level [26]. From phylogenetic analysis of *ITS* and *gpd* sequences on the taxonomy of *Stemphylium*, it was reported that *S*. *callistephi* and *S*. *solani* were placed as phylogenetically distinct groups from the other species, whereas, *S*. *vesicarium, S*. *herbarum, S*. *alfalfae, S*. *tomatonis*, and *S*. *sedicola* were grouped in the same clade, as these could not be differentiated on the basis of molecular data [24,26,27]. Differentiation of two closely related species of *Stemphylium*, i.e., *S. vesicarium* and *S. botryosum*, based on morphological characters is very difficult due to the strong phenotypical similarities. However, the two species were easily differentiated based on a 3 kb intron present in the *S*. *botryosum* cytochrome b region but not in *S*. *vesicarium* by analyzing sequence of protein coding gene of cytochrome b [28]. 

## 4. Population Genetic Structure

Selection of the best loci for genetic and population diversity analysis is the prerequisite for the phylogenetic description. Combined analysis of *ITS*, *gpd*, and *Calmodulin* loci was carried out to construct a phylogenetic overview of the genus *Stemphylium* [27]. An attempt has been made to infer phylogenetic informativeness of seven commonly used protein coding genes, namely *ITS, gpd, calmodulin, 28S rRNA, ATPase, elongation factor-1 alpha* (*Ef-1 alpha*),and *histidine kinase* from already available *Stemphylium* sequence information obtained from NCBI nucleotide database (Supplementary Table S1).Population diversity indices such as numbers of segregating sites (s), haplotype number (h), haplotype diversity (Hd), nucleotide diversity (π), and average number of pairwise nucleotide differences within population (K), were estimated using DnaSP 6 Software [29]. Further, to test deviations from neutral molecular evolution, Tajima’s D- and Fu’s Fs-tests were carried out by Arlequin version 3.1 [30] through the generation of random samples under the hypothesis of selective neutrality and population equilibrium. Moreover, for obtaining maximum evolutionary information among the closely related species of *Stemphylium* based on substitutions and insertion-deletion (indels) analyses using distance-based framework, the r-package SIDIER [31] was employed to reconstruct the evolutionary relationship. The analysis of molecular variance (AMOVA) was also performed using concatenated gene sequences (*ITS*, *gpd*, and *calmodulin*) to compare species of *Stemphylium* considered as populations. AMOVA calculations were conducted in Arlequin version 3.1 [30]. AMOVA partitioned total variance into among populations and within populations and this statistical analysis is considered as an effective tool to define population structure and degree of genetic differentiation. Bayesian analysis of the genetic structure in *Stemphylium* was performed using BAPS package version 6 (http://www.helsinki.fi/bsg/software/BAPS/) from concatenated *ITS*, *gpd*, and *calmodulin* gene sequences, which treat nucleotide frequencies and the number of genetically diverged groups in the population as random variables and provides the most appropriate population structure with the optimum number of subgroups.

*EF-1 alpha* and *calmodulin* exhibited higher values of diversity statistics such as K, *π*, Hd, and *calmodulin*, representing the maximum number of haplotypes, though with a smaller number of sequences (Table 2). *EF-1 alpha* exhibited high genetic diversity. These two loci were followed by *ATPase* and *gpd*, for which almost all the parameters (except number of haplotypes in *ATPase*) are higher. *ITS* has relatively low nucleotide and average haplotype diversity. However, this locus is universally considered as an important taxonomical unit because of high conservation and evolutionary trend of changes (A.D., unpublished data). 

**Figure 4:**
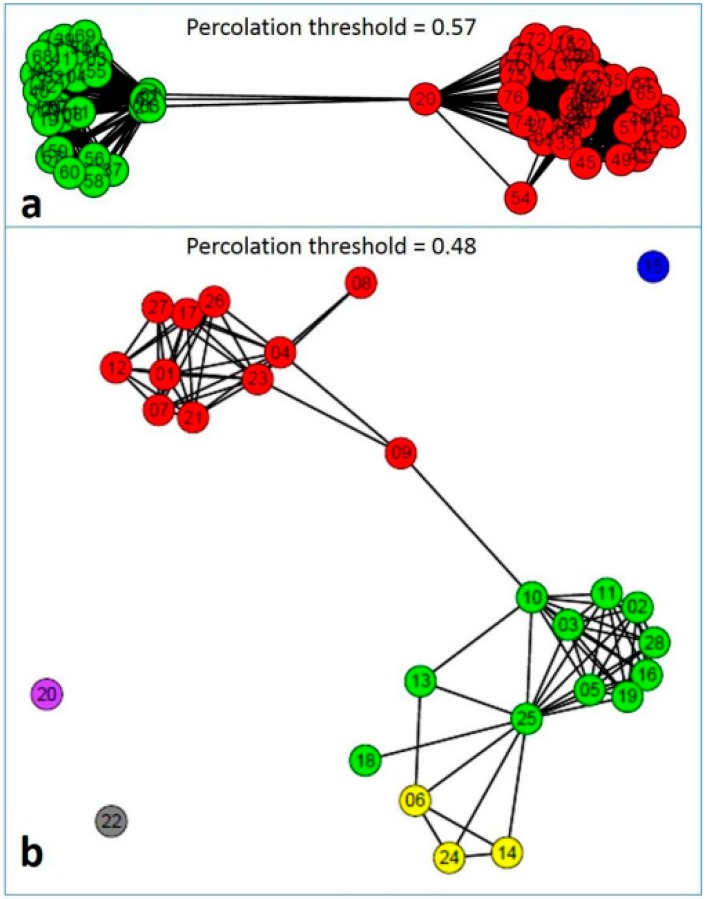
Percolation network generated combining distance matrices of indels, and substitutions drawn by the SIDIER package. The network was generated connecting distances lower than the estimated percolation threshold (depicted in the figure). Groups are represented in different colors (A.D., unpublished data). (**a**). Relationships among 76 haplotypes obtained from the analysis of concatenated *ITS*, *gpd*, and *calmodulin* sequences of 157 *Stemphylium* strains involving 28 species. Haplotypes were diversified into two distantly related groups. The first group is described in green color, containing species (*S*. *amaranthi*, *S*. *beticola*, *S*. *canadense*, *S*. *chrysanthemicola*, *S*. *drummondii*, *S*. *simmonsii*, *S*. *halophilum*, *S*. *loti*, *S*. *lycii*, *S*. *paludiscirpi*, *S*. *sarciniforme*, *S*. *trifolii* and *S*. *triglochinicola*) and another group in red color (*S*. *armeriae*, *S*. *astragali*, *S. botryosum*, *S*. *callistephi, S*. *drummondii*, *S*. *eturmiunum*, *S*. *gracilariae*, *S*. *ixeridis*, *S*. *lancipes*, *S*. *lucomagnoense*, *S*. *lycopersici*, *S*. *majusculum*, *S*. *novae-zelandiae*, *S*. *solani*, *S*. *symphyti* and *S*. *vesicarium*). (**b**). Relationship among 28 species of *Stemphylium*. Except three distantly related species (15) in blue: *S. loti*, (20) in Dark Orchid: *S. novae-zelandiae* and (22) in grey: *S. sarciniforme*), other species were delineated into three groups.

**Figure 5:**
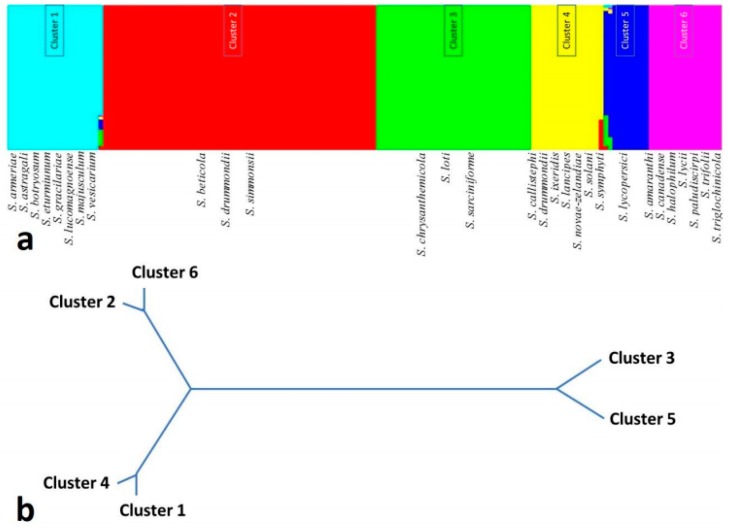
Bayesian inference of genetic structure of the 28 species of *Stemphylium* differentiated two haplotypic groups into six clusters (157 strains containing 57 haplotypes based on concatenated *ITS*, *gpd*, and *calmodulin* sequences) analyzed through BAPS package version 6 (A.D., unpublished data). (**a**). All the species were diversified into six groups with existence of admixture in some of the species as indicated by color variegation, obtained through admixture analysis. (**b**). Phylogenetic relationship using UPGMA clustering method among the six BAPS groups.

Other diversity parameters such as Tajima’s and Fu’s neutrality tests elucidate evolutionary characteristics of the locus. Both values for *28S rRNA* gene are negative, indicating a high level of population expansion with excess number of alleles. Tajima’s D value for *ITS* sequences is also negative, indicating the usefulness of this gene for taxonomic analysis (A.D., unpublished data). Other moderate-to-less sequenced loci, such as *ATPase*, *EF-1 alpha*, and *histidine kinase* have high Fu’s Fs values, indicating less conservation and high allelic diversity. Both parameters for *gpd* and *calmodulin* are not significant. Analysis of the haplotype and species level divergence of *Stemphylium* with concatenated aligned *ITS*, *gpd*, and *calmodulin* gene sequences utilizing r-package SIDIER employing both indel and substitution indicated interesting phenomena. A percolation network drawn from the combined distance has diversified 76 haplotypes of the 28 species of *Stemphylium* into two groups (Figure 4). The green colored group contains 13 species while the red haplotypic group contains 16 species. In contrast, the percolation network based on species considered as population has differentiated 28 species into three major groups and three groups containing one isolate each (Figure 4). Bayesian analysis again divided the *Stemphylium* species complex into six groups (Figure 5); the red haplotypic group in Figure 5 is subdivided into Cluster 1, 4, and 5, whereas green haplotypic grouped into Cluster 2, 3, and 6. It indicates some species have shared genetic material and are either evolved from, or still admixing to, another taxonomic unit. According to AMOVA results (Table 3), species of *Stemphylium* were significantly diverged from each other (96.79% among population variation) with higher and significant Wright’s *F*-statistics (F_ST)_ value (0.97). Little progress has been made with respect to molecular diversity of *S. botryosum*, and only few sequences could be retrieved from genomic DNA database for a geographical diversity analysis of the species. 

## 5. Epidemiology

*Stemphylium* spp. can survive on infected plant debris, seeds, and in soil. In Canada, it was reported that *S*. *botryosum* has an ability to survive long winters and to sporulate in hot summers [5]. The development of pseudothesia on plant debris depends on environmental conditions. Secondary spread occurs through air borne conidia.

Reports are unavailable regarding histological studies for determining the sequence and form of pathogen virulence in lentil *Stemphylium* pathosystems. Reviewing the available reports in other pathosystems, it can be stated that airborne conidia germinate on leaf surfaces in the presence of a thin film of moisture. Generally, the penetration of the germ tube occurs through stomata as well as directly through the epidermis in rape [32]. Penetration through stomata is also affected by host resistance but is governed by environmental factors as reported for *S*. *botryosum* in alfalfa [21]. 

Disease incidence and its development in lentils are influenced by different environmental factors like temperature, relative humidity (RH), rainfall, number of cloudy days, and wetness period [5,9,10]. Temperature and moisture are primary environmental factors affecting conidial germination of *S*. *botryosum* and play important role in disease incidence. An average mean temperature of 18 ± 2 °C and morning RH of 85%–90% are favorable for the appearance, development, and spread of the disease, while an afternoon RH of more than 50% is essential in Indian conditions [15]. The other important factor in determining the appearance and development of the disease is the number of cloudy and foggy days, which is between 30 and 45 days in favorable years and between 17 and 23 days in unfavorable years. In Bangladesh, 97% RH, cloudy weather, and temperature of 20–22 °C favors disease development [33]. Under controlled conditions, conidia of *Stemphylium* germinate at temperatures ranging from 5 °C to 30 °C [5]. *S*. *botryosum* initiated infection on lentils when the night temperature remained above 8 °C with average day temperatures above 22 °C and the relative humidity in the plant canopy exceeded 95%. In a recent study the minimum latent period in lentils was 48 h at the ideal temperature of 25–30 °C under controlled conditions. It increased with decreases in temperature and wetness period [5].

## 6. Symptoms and Disease Assessment

**Figure 6:**
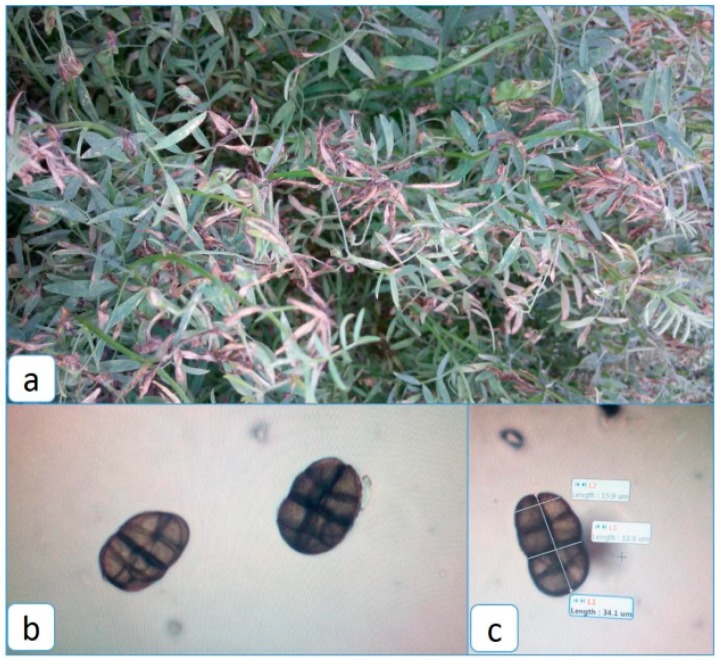
Symptomology and microscopy of *Stemphylium botryosum*, incitant of blight in lentils. (**a**). Symptom of Stemphylium blight in the foliage of lentils at reproductive stage. (**b**). Microphotograph of conidia of *Stemphylium botryosum* isolated from lentils. (**c**). Measurement of conidial morphological parameters.

Disease symptoms have been well characterized in South Asia where *S. botryosum* has caused great devastation to the lentil crop (Figure 6). The pathogen attacks the crop in the early pod setting stage and symptoms appear as pin-headed light brown to tan colored spots on the leaflets which later enlarge, covering the leaf surface within 2 to 3 days [34]. A blighted dull yellow appearance is observed in infected foliage and branches. Defoliation occurs rapidly, leaving the branches with terminal leaves. The stems and branches also bend down, dry up, and gradually turn ashy white, but the pods remain green. Pedicels and flowers can also be infected, the latter resulting in flower abortion [5]. Symptoms are prominent in the upper canopy, but entire plants can be blighted under severe infestation. Significant leaf drop, loss of biomass and seed yield, and a reduction in seed size can also occur. Infected seeds are often stained and can have low germination rates. White mycelia growth can also be observed on the infected stems. Sometimes it is suspected that SB has not been correctly identified in the field, as the lesions closely resemble those of Ascochyta blight [6]. 

Different descriptive scales have been used by several workers based on some qualitative and quantitative characters. A semiquantitative 0–10 scale has been suggested for scoring disease severity [35]. However, the most common disease rating scale is 1–9 [14]. Field screening against *Stemphylium botryosum* has been standardized by several workers [36,37,38,39]. For creating artificial epiphytotic condition, the testing materials are inoculated during the flowering stage with mycelial suspension (2 × 10^5^ conidia mL^−1^) in the evening on cloudy day. Following inoculation, plant materials are subjected to sprinkler irrigation to maintain 80%–85% of leaf wetness for creating a congenial environment for germination of conidia. 

A controlled environment facilitates the reliable screening of lentil genotypes against SB as in field conditions, because ambiguities are created due to presence of the closely related genus *Alternaria* spp. Therefore, controlled screening techniques have been standardized for the screening of lentil genotypes against SB [5,38]. Since *S. botryosum* does not sporulate well on ordinary synthetic media, the large-scale conidia production of *S. botryosum* isolates of lentils has not been optimized to allow large pathogenicity studies. 

## 7. Secondary Metabolites and Pathogenicity

Some strains of *Stemphylium* have been shown to produce a wide range of secondary metabolites, of which many probably play a role during host plant infection as phytotoxins or host-specific toxins [40]. It was reported that pathogenicity of *S. botryosum* on rape is associated with production of the phytotoxin stemphol [32]. Culture filtrates of some isolates of *S. vesicarium* have been shown to be pathogenic to either European pear cultivars or Japanese pear cultivars, but never both [41]. The culture filtrates contain host-specific toxins (SV-toxins I and II) that have not been fully described yet [42]. Two endophytic strains of *S. globuliferum* also produced alterporriols H and K, altersolanol L, stemphypyrone [43], alterporriols D and E, altersolanol A, altersolanols B and C, and macrosporin [44], while an another endophytic strain of *S. botryosum* produced altersolanol A, curvularin, dehydrocurvularin, macrosporin, and stemphyperylenol [45]. A strain of *S. herbarum* produced alterporriols D-G and altersolanol A [46]. Recently, it has also been shown that *Stemphylium* metabolites have biological activities, such as cytotoxic and antibacterial effects [43,44] that may be of interest to the pharmaceutical industry. Metabolite profiling of *Stemphylium* spp. has also been studied by several workers [47]. However, it has some limitations of stopping sporulation and losing metabolite production of a few strains when cultures are grown repeated times in artificial media for a long time. 

## 8. Disease Management

### 8.1. Host Plant Resistance (HPR)

SB resistance is associated with variation in anatomical features of the host plant. It was observed that lentil cultivars with thicker cuticle and epidermal cell layers, fewer stomata, and large numbers of epidermal hairs exhibited SB resistance [48]. These anatomical features act as a mechanical barrier for penetration and further entry of the *S*. *botryosum* through hyphae within lentils. Generally, *S*. *botryosum* enters through stomata and forms substomatal bulbous mycelium within the host which is influenced by relative pathogen virulence and environmental factors [21].

Studies regarding genetics and inheritance of SB resistance are still in the amorphous stage with lots of ambiguities in the reports regarding the inheritance pattern. Initial reports considering Bulgarian lentil cultivars revealed complex resistance towards SB. Recombinant Inbred Line population (RIL) of lentils developed from a cross between Barimasur-4 × CDC Milestone as resistant and susceptible parents revealed quantitative inheritance. Another study was attempted with F_1_, back cross population and RILs obtained from a cross between a resistant line, ILL-6002, and a susceptible line, BM-1 (ILL-5888), to determine the genetics of resistance as well as number of genes and quantitative trait loci (QTL) associated with disease resistance. The presence of dominant genes, along with significant additive and epistatic gene action towards the QTLs governing resistance, was detected [37]. However, the genetics of resistance should also be determined in other genetic backgrounds to gain further insight into genetic resistance. On the contrary, six reports are available regarding the genetics of SB resistance in other host plants which are more precise and conclusive. Therefore, concerted efforts are pertinent for generating definitive information regarding SB resistance in lentil. 

Several studies have been conducted considering cultivated and wild species of lentil for searching out resistant sources for SB (Table 4). Lentil cultivar, Precoz (ILL-4605), has been identified as resistant to *S. botryosum*. The study conducted in Bangladesh confirmed the lentil cultivar, Barimasur-4, is resistant to *S. botryosum*. Comprehensive screening of lentil genotypes against SB in Bangladesh revealed a variable genotypic response with increment of sensitivity of the cultivars with the increase in their growth stage [49]. In a different study by Crimson and Eston, ILL-4605-2 and ILL-8008 were identified as good resistant sources [35]. Under artificial epiphytotic conditions, 15 entries were detected as moderately resistant [50]. Diversity analysis with SSR markers identified VL-151 as most diverse amid the moderately resistant cultivars and recommended for utilization as a parent in the resistance breeding program in lentils [51]. Six genotypes, viz., ILL-0426, ILL-0427, ILL-0215, ILL-6408, ILL-0133, and ILL-0379, were also identified as resistant sources for future exploitation in a lentil breeding program in Australia [14].

### 8.2. Integrated Disease Management

Disease caused by members of *Pleospora*, like *Alternaria* and *Stemphylium*, is difficult to manage because of its capacity to produce huge amounts of secondary inoculum in a short period under favorable environmental conditions. Under in vivo conditions, sporulation of *Alternaria* and *Stemphylium* is affected by various external factors such as light, temperature, nutrients, and photo periods [52]. For effective control, farmers use several fungicidal sprays often from early growing season until maturity. Integrated disease management (IDM) by involving cultural, physical, biological, and chemical tools is the best option for managing SB, particularly in high epidemic areas like Bangladesh and Nepal. There is no region-specific IDM package for SB in lentils. Modification of sowing time, crop rotation with non-hosts, field sanitation, seed treatment by physical and chemical means, application of effective biocontrol agents, use of resistant varieties, and finally rotational use of some protectant and curative fungicides having different modes of action gives best management of SB. Studies conducted in South Asia confirmed that early sowing of lentils before the middle of November drastically reduced Percent Disease Index (PDI) without compromising yield [10,12]. *S. botryosum* invasions in lentils can be efficaciously accomplished through applications of botanical extracts. The extract from *Acorus calamus* and *Zanthozylum armatum* significantly suppressed the colony of *S*. *botryosum* and was thereby recommended for managing SB [53]. In the absence of resistant cultivars, strategic application of fungicides, viz., chlorothalonil, mancozeb, tebuconazole, procymidone, and iprodione, is effective in controlling SB in lentils [54,55,56]. However, the labels claim that the fungicides in lentils are an issue in some countries. Comprehensive studies on management of SB revealed the urgency of forecasting model for prediction of disease epidemics followed by judicial fungicidal application for proper disease management [56,57,58]. Different predictive models such as TOM-CAST [56], FAST [57], and BSPcast [58] have been used for the prediction of disease initiation by *Stemphylium* spp. on many hosts and scheduling of fungicidal spray based on temperature and leaf wetness periods. Stempedia, a weather-based model was developed to understand the risk of SB disease in Bangladesh [59]. Sowing date, date of first flowering, and daily weather variables, like maximum temperature and sunshine hours, are the important input parameters of this model. The model further estimates the financial gain/loss with or without disease control (by fungicide application) scenarios considering crop losses, costs of the fungicide, and its application. Therefore, such predictive models need to be developed for each major lentil growing region of the world.

## 9. Future Outlook

**Figure 7:**
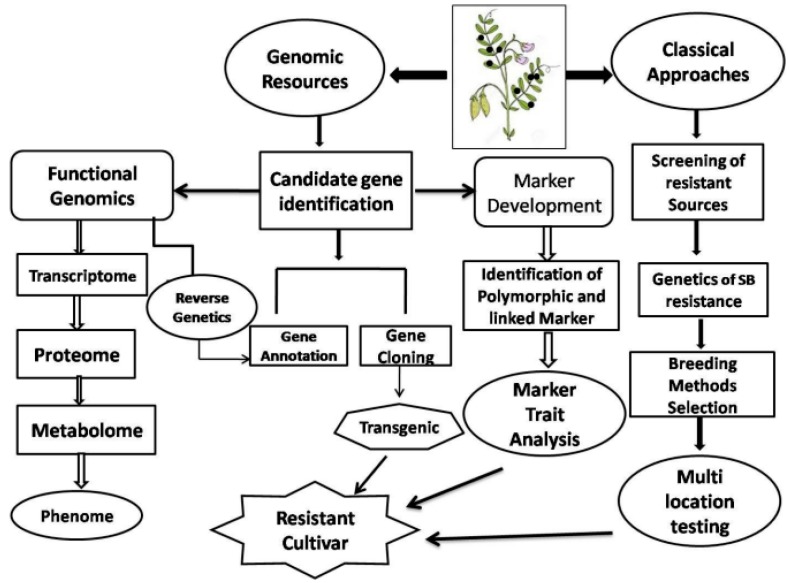
An overview of breeding tools and ‘omics’ approaches for the development of SB resistant cultivars in lentils. The proposed model depicts how the different breeding approaches can be deployed for developing SB resistance in lentils.

SB has emerged as a serious threat to lentil production globally. Given the threat that this disease may pose to lentil growing areas in future, concerted research efforts are required to understand the biology, pathogenicity, and genetic basis of resistance towards integrated disease management. Details regarding the histopathological study to draw conclusion about the infection process in lentils is missing, though in other pathosystems extensive investigations have been made. Knowledge regarding the defense mechanism of the host is also missing in lentil *Stemphylium* pathosystems for devising suitable disease management strategy. A genetic basis of different host associations based on different *S*. *botryosum* isolates needs to be established. The genetics of SB resistance in lentils are still in their infancy, therefore immediate attention for comprehensive research is needed using conventional, molecular, and ‘omics’ tools (Figure 7). Construction of linkage maps utilizing crop wild relatives (CWR) viz., *L*. *ervoides* or *L. lamottei*, is urgent for refining towards the QTL mapping of SB resistance in lentils. Functional genomics and ‘omics’ tools can open new perspectives through elucidating the candidate genes and their ontology, transcripts variables, proteins, and metabolites catalogue, mediating the complex defense mechanisms in relation with lentil *Stemphylium* pathosystem. Multilocation testing for the identification of durable resistance is a prerequisite for a resistance breeding program against SB. With the introduction of SB into South Asia, where the disease is likely to cause significant losses in the future due to the existence of a congenial environment and susceptible varieties, a detailed SB risk analysis is urgently needed. As *S*. *botryosum* is genetically distinct and highly diverse with the broad host range, it has important implications for quarantine and biosafety regulations to avoid additional spread of the pathogen to disease-free countries. Genomic monitoring is essential to track the evolution of *S*. *botryosum* in endemic areas of Bangladesh and South Asia. There is a need to improve the effectiveness of new chemicals for controlling the disease. The development of forecasting and prediction models is justified as an integral component of IDM of SB resistance in lentils. 

## Figures and Tables

**Table 1:** Morphological characters of related genera of *Stemphylium.*

Genera	*Alternaria*	*Stemphylium*	*Pithomyces*	*Epicoccum*	*Ulocladium*
**Colony character**	Olivaceous to gray to black woolly colonies	Velvety to cottony brown or black colony	Brown to black in color	Colony fast growing, with a strong yellow to orange-brown diffusible pigment.	Distinctive yellow to orange-brown color colony with brown diffusing pigment
**Conidium shape**	Large, dark muriform with beak	Large, dark muriform	Large, dark muriform	Large, dark muriform	Large, dark verrucose muriform
**Conidium formation**	Conidia formed in chains or singly	Conidia formed singly	Conidia formed singly	Conidia formed singly on densely compacted, non-specialized, determinant	Conidia formed singly
**Conidium arrangement**	Lacks percurrent proliferation (Conidia produced through nodes on conidiophores	Percurrent proliferation present	Lacks percurrent proliferation and geniculate conidiophores	Conidial production restricted to sporodochia areas	Conidia formed in a sympodial fashion from geniculate conidiophores
**Conidiophore**	Erect, septate, and geniculate	Short, arise singly or in whorls, septate and swollen at the apex.	Short, peg like lateral branches from the vegetative hyphae	Nonspecialized, determinant; branches repeatedly and visible as dense masses in sporodochia	Simple or branched, smooth, strongly geniculate

Source: Modified from Woudenberg et al., 2013 [25].

**Table 2:** Overview of the genetic data of various loci.

Locus	#ind	NS	*s*	K	*π*	#h	Hd	Fs	D (*p* Value)
*ITS*	157	518	53	6.44	0.0133 ± 0.0008	27	0.85 ± 0.02	−4.493 (0.21)	**−1.215** (0.09)
*gpd*	157	516	151	24.57	0.0495 ± 0.0017	43	0.92 ± 0.02	3.368 (0.82)	−0.180 (0.50)
*Calmodulin*	157	664	206	42.43	0.0704 ± 0.0019	49	0.95 ± 0.01	8.950 (0.94)	0.476 (0.74)
*28S rRNA*	22	796	50	4.91	0.0062 ± 0.0045	7	0.67 ± 0.09	**−21.560** (0.00)	**−2.535** (0.00)
*ATPase*	47	684	183	38.38	0.0598 ± 0.0069	22	0.93 ± 0.02	**−17.938** (0.00)	−0.308 (0.45)
*EF-1*	51	861	323	47.97	0.0786 ± 0.0149	21	0.93 ± 0.02	**−11.313** (0.00)	−1.085 (0.12)
*Histidine kinase*	9	1187	4	1.39	0.0007 ± 0.0001	5	0.89 ± 0.07	**−10.848** (0.00)	−0.229 (0.41)

(Source: A.D., unpublished data). NS, Number of sites. #ind, number of individuals sequenced at locus. *s*, number of polymorphic sites. K, average number of nucleotide differences between sequences. *π*, nucleotide diversity. #h, number of haplotypes. Hd, haplotype diversity. Fs, Fu’s Fs (significant values at *p* < 0.02 are in bold). D, Tajima’s D (significant values at *p* < 0.1 based on 1000 permutations in Arlequin in bold).

**Table 3:** AMOVA of various *Stemphylium* species considered as populations.

Sources of Variation	Sum of Squares	Variance Components	Percentage Variation
Among populations	7003.487	49.68865	96.78650
Within populations	212.819	1.64976	3.21350
Total	7216.306	51.33841	
F_ST_	0.96786 (*p* < 0.0001)		

(Source: A.D., unpublished data).

**Table 4:** Sources of resistance to Stemphylium blight in lentil germplasm.

Serial	Genotypes	Remark	References
1.	Barimasur-4	Resistant	[33]
2.	Eston and IG-72815	Resistant	[36]
3.	10/P8406-122, FLIP-92- 52LX, LR-9-135, LR-9-130, LR-9-179, LR-9-69, LR-9-69, LR-9-100, LR-9-118, LR-9-28, LR-9-25, Procoz, LR-9-57, LR-9-107, LR-9-105, LR-9-48, LR-9-62, LR-9-25, 10/P11X955-135, 10/P2 FLIP-92-52LX955-167(4), and 10/P8405-23	Resistant	[60]
4.	ILL-7164, ILL-6458, ILL-1704, ILL-9927, ILL-8006(BM-4), ILL-1672, X94s43, ILL-2573, ILL-9992, ILL-6025, Aarial, ILL-8093, ILL-9976, ILL-6256, IL-1, ILL-6818, ILL-2700-1, X94s29, ILL- 9931, ILL-9996, ILL-5787, and ILL -8191	Moderately Resistant	[61]
5.	IG-72803, IG-116033, L-01-827, IG-72548, IG-72551, IG-72553, IG-72557, IG-72713, IG-72843, IG-136645, IG-72829, IG-72643, IG-72606, IG-72537, IG-72552, and IG-110809	Resistant	[38]
6.	BLX-06004-12, BLX-06004-2, and BLX-05001-6	Moderately resistant	[62]
7.	LL-1370, VL-151, LL-1375, RLG-195, L-4727, L-4769, LL-1397, DL-14-2, VL-526, VL-126, RKL- 14-20, IPL-334, L-4710, PL-210, and Precoz	Moderately resistant with 30% of foliage affected	[50]
8.	P-3235, LL-1122, and ILL-10832	Immune	[39]
9.	L01-827A and IG-72815	*Lens ervoides* accessions showing multiple resistance	[63]
10.	ILL-0426, ILL- 0427, ILL-0215, ILL-6408, ILL-0133, ILL-0379, ILL-0365, and ILL-0192	Resistant to moderately resistant	[14]
11.	RL-13, RL-21, ILL-6468, ILL-9996, ILL-6024, ILL-6811, ILL-7164, Arun, and Maheswar Bharti,	Multiple Resistant	[64]
12.	BD-3921, BD-3930, BD-3931, and BARI Masur-7	Highly Resistant	[65]

## Supplementary Materials

The following are available online at https://www.mdpi.com/2076-0817/8/4/225/s1, Table S1: Accessions of ITS, *gpd, calmodulin*, *28S rRNA*, *ATPase*, *EF-1 alpha* and *histidine kinase* genes of various species of *Stemphylium* used in phylogeny studies as obtained from NCBI Nucleotide database (https://www.ncbi.nlm.nih.gov/nucleotide/).
