# Supplementary material for: Current Knowledge on Pathogenicity and Management of Stemphylium botryosum in Lentils (Lens culinaris ssp. culinaris Medik)
Source: Pathogens. 2019 Nov 8;8(4):225. doi: 10.3390/pathogens8040225 (PMC6963855; doi:10.3390/pathogens8040225)
Supplement: Supplementary file 1 [file pathogens-08-00225-s001.pdf]

Supplementary Table 1: Accessions of *ITS*, *gpd*, *calmodulin*, *28S rRNA*, *ATPase*, *EF-1 alpha* and *histidine kinase* genes of various species of *Stemphylium* used in phylogeny studies as obtained from NCBI Nucleotide database (https://www.ncbi.nlm.nih.gov/nucleotide/)

Accession	Species	Strain/Isolate	Country
***ITS* gene**			
KU850503	*S. amaranthi*	CBS 124650	China
KU850504	*S. amaranthi*	CBS 124651	China
KU850505	*S. amaranthi*	CBS 124746	China
KU850506	*S. amaranthi*	CBS 124750	Chile
KU850507	*S. amaranthi*	CBS 124753	China
KU850508	*S. amaranthi*	CBS 124984	China
KU850509	*S. amaranthi*	CBS 124985	China
KU850510	*S. amaranthi*	CBS 124989	New Zealand
KU850510	*S. amaranthi*	CBS 136589	New Zealand
KU850511	*S. armeriae*	CBS 338.73	UK
KU850512	*S. astragali*	CBS 116583	Japan
KU850514	*S. beticola*	CBS 116599	Canada
KU850515	*S. beticola*	CBS 133512	Canada
KU850516	*S. beticola*	CBS 133892	USA
KU850517	*S. beticola*	CBS 136590	New Zealand
KU850518	*S. beticola*	CBS 136699	USA
KU850519	*S. beticola*	CBS 137492	USA
KU850520	*S. beticola*	CBS 141024	Netherlands
KU850521	*S. beticola*	CBS 141025	Netherlands
KU850522	*S. beticola*	CBS 141026	Netherlands
KU850513	*S. beticola*	CBS 378.54	Canada
KU850523	*S. beticola*	GV11-196a1-3	Netherlands
KU850524	*S. beticola*	GV12-275a1	Netherlands
KU850525	*S. beticola*	GV12-276a1	Netherlands
KU850526	*S. beticola*	GV12-287a1	Netherlands
KU850527	*S. beticola*	GV12-336a1	Netherlands
KU850528	*S. beticola*	GV12-356a1	Netherlands
KU850529	*S. beticola*	GV12-367a1	Netherlands
KU850530	*S. beticola*	GV12-368a1	Netherlands
KU850531	*S. beticola*	GV12-403a1	Netherlands
KU850532	*S. beticola*	GV13-425a1	Netherlands
KU850533	*S. beticola*	GV13-436c2	Netherlands
KU850534	*S. beticola*	GV14-693a1	UK
KU850535	*S. beticola*	IFZ2013-024	Germany
KU850535	*S. beticola*	IFZ2013-035	Germany
KU850537	*S. beticola*	IFZ2014-020	Germany
KU850538	*S. botryosum*	CBS 116596	USA
MH859208	*S. botryosum*	CBS 714.68	Canada

MH206172	*S. botryosum*	M-14	China
MH206170	*S. botryosum*	M-2	China
MH206171	*S. botryosum*	M-4	China
MH856744	*S. callistephi*	CBS 527.50	USA
AF229482	*S. callistephi*	EEB 1055	Unknown
KU850641	*S. canadense*	CBS 116602	Canada
KU850642	*S. canadense*	CBS 118081	Canada
KU850640	*S. chrysanthemicola*	CBS 117255	New Zealand
GQ395365	*S. drummondii*	CBS 346.83	Germany
KU850632	*S. drummondii*	CBS 716.68	USA
KU850541	*S. eturmiunum*	CBS 109845	New Zealand
KU850542	*S. eturmiunum*	CBS 122124	Greece
KU850543	*S. eturmiunum*	CBS 122641	France
KU850544	*S. eturmiunum*	CBS 124652	China
KU850545	*S. eturmiunum*	CBS 133528	India
KU850546	*S. eturmiunum*	CBS 138495	China
KU850540	*S. eturmiunum*	CBS 668.80	Greece
KU850550	*S. gracilariae*	CBS 115179	Spain
KU850551	*S. gracilariae*	CBS 115180	Spain
KU850552	*S. gracilariae*	CBS 125060	China
KU850548	*S. gracilariae*	CBS 273.55	Unknown
KU850547	*S. gracilariae*	CBS 308.36	USA
MH862230	*S. gracilariae*	CBS 482.90	Israel
MH860692	*S. halophilum*	CBS 337.73	UK
KU850554	*S. halophilum*	CBS 410.73	UK
KU850590	*S. ixeridis*	CBS 124748	China
KU850594	*S. lancipes*	CBS 101217	New Zealand
KU850595	*S. lancipes*	CBS 116584	New Zealand
KU850596	*S. lancipes*	CBS 133314	USA
MH857374	*S. loti*	CBS 407.54	USA
KU850629	*S. lucomagnoense*	CBS 116601	Switzerland
KU850598	*S. lycii*	CBS 115192	Portugal
KU850599	*S. lycii*	CBS 116582	USA
KU850600	*S. lycii*	CBS 124982	China
KU850601	*S. lycii*	CBS 125240	China
KU850602	*S. lycii*	CBS 125241	China
MH862998	*S. lycopersici*	CBS 116585	New Caledonia
KU850608	*S. lycopersici*	CBS 116587	Dominican Republic
KU850609	*S. lycopersici*	CBS 120325	China
KU850610	*S. lycopersici*	CBS 120326	China
KU850611	*S. lycopersici*	CBS 122639	China
MH863236	*S. lycopersici*	CBS 122803	China
KU850613	*S. lycopersici*	CBS 123008	China
KU850614	*S. lycopersici*	CBS 124980	China

KU850615	*S. lycopersici*	CBS 124981	China
KU850616	*S. lycopersici*	CBS 124983	China
KU850617	*S. lycopersici*	CBS 135778	New Zealand
KU850606	*S. lycopersici*	CBS 321.87	Senegal
KU850603	*S. lycopersici*	CBS 333.73	Netherlands
KU850604	*S. lycopersici*	CBS 436.76	Indonesia
KU850605	*S. lycopersici*	CBS 463.78	Peru
MF508967	*S. lycopersici*	LJ1609270201	China
KU850619	*S. majusculum*	CBS 133424	USA
MH859209	*S. majusculum*	CBS 717.68	USA
KU850630	*S. novae-zelandiae*	CBS 138157	New Zealand
KU850631	*S. novae-zelandiae*	CBS 138295	New Zealand
MH862838	*S. paludiscirpi*	CBS 109842	USA
KU850591	*S. sarciniforme*	CBS 110049	Iran
KU850623	*S. sarciniforme*	CBS 116579	USA
KU850592	*S. sarciniforme*	CBS 116581	Iran
KU850624	*S. sarciniforme*	CBS 133723	USA
KU850593	*S. sarciniforme*	CBS 136810	Iran
KU850625	*S. sarciniforme*	CBS 138345	New Zealand
KU850621	*S. sarciniforme*	CBS 335.33	USA
KU850622	*S. sarciniforme*	CBS 364.49	USA
KU850633	*S. simmonsii*	CBS 116598	Canada
KU850634	*S. simmonsii*	CBS 116603	Canada
KU850635	*S. simmonsii*	CBS 116604	Canada
KU850636	*S. simmonsii*	CBS 133515	Canada
KU850637	*S. simmonsii*	CBS 133518	Canada
KU850638	*S. simmonsii*	CBS 133894	USA
KU850639	*S. simmonsii*	CBS 134496	Australia
KU850632	*S. simmonsii*	CBS 716.68	USA
KU850627	*S. solani*	CBS 116586	USA
KU850628	*S. solani*	CBS 118082	USA
MH857375	*S. solani*	CBS 408.54	USA
KY883858	*S. symphyti*	CBS 115268	USA
KU850644	*S. symphyti*	CBS 118796	New Zealand
KU850645	*S. symphyti*	CBS 138069	New Zealand
KU850646	*S. symphyti*	CBS 138070	New Zealand
KU850647	*S. trifolii*	CBS 116580	USA
MH859210	*S. triglochinicola*	CBS 718.68	UK
KU850571	*S. vesicarium*	CBS 109843	New Zealand
MH862840	*S. vesicarium*	CBS 109844	USA
KU850573	*S. vesicarium*	CBS 115182	South Africa
KU850574	*S. vesicarium*	CBS 115204	Portugal
KU850575	*S. vesicarium*	CBS 122640	China
KU850576	*S. vesicarium*	CBS 123005	China

KU850577	*S. vesicarium*	CBS 123803	China
KU850578	*S. vesicarium*	CBS 124279	Denmark
KU850579	*S. vesicarium*	CBS 124747	China
MH863402	*S. vesicarium*	CBS 124749	China
KU850581	*S. vesicarium*	CBS 124751	China
KU850582	*S. vesicarium*	CBS 124752	China
KU850583	*S. vesicarium*	CBS 125242	China
KU850584	*S. vesicarium*	CBS 133474	USA
KU850585	*S. vesicarium*	CBS 133737	Australia
KU850586	*S. vesicarium*	CBS 133905	USA
KU850587	*S. vesicarium*	CBS 133914	USA
KU850588	*S. vesicarium*	CBS 138138	Netherlands
KU850555	*S. vesicarium*	CBS 155.24	Unknown
KU850561	*S. vesicarium*	CBS 156.45	Netherlands
KU850556	*S. vesicarium*	CBS 157.24	Unknown
KU850557	*S. vesicarium*	CBS 184.25	UK
MH861935	*S. vesicarium*	CBS 191.86	India
KU850568	*S. vesicarium*	CBS 192.86	Australia
KU850567	*S. vesicarium*	CBS 205.82	Netherlands
KU850558	*S. vesicarium*	CBS 273.31	Unknown
KU850559	*S. vesicarium*	CBS 274.31	Unknown
KU850560	*S. vesicarium*	CBS 307.36	Tunisia
KU850569	*S. vesicarium*	CBS 311.92	Netherlands
KU850562	*S. vesicarium*	CBS 322.49	Netherlands
KU850564	*S. vesicarium*	CBS 368.59	Denmark
KU850563	*S. vesicarium*	CBS 370.51	Netherlands
KU850566	*S. vesicarium*	CBS 406.76	Germany
KU850570	*S. vesicarium*	CBS 486.92	Netherlands
KU850565	*S. vesicarium*	CBS 715.68	Canada
KU850589	*S. vesicarium*	GV11-355a1-2	Netherlands
MH206173	*S. vesicarium*	M-6	China
***gpd* gene**			
KU850650	*S. amaranthi*	CBS 124650	China
KU850651	*S. amaranthi*	CBS 124651	China
KU850652	*S. amaranthi*	CBS 124746	China
KU850653	*S. amaranthi*	CBS 124750	Chile
KU850654	*S. amaranthi*	CBS 124753	China
KU850655	*S. amaranthi*	CBS 124984	China
KU850656	*S. amaranthi*	CBS 124985	China
KU850657	*S. amaranthi*	CBS 124989	New Zealand
KU850657	*S. amaranthi*	CBS 136589	New Zealand
KU850658	*S. armeriae*	CBS 338.73	UK
KU850659	*S. astragali*	CBS 116583	Japan
KU850661	*S. beticola*	CBS 116599	Canada

KU850662	*S. beticola*	CBS 133512	Canada
KU850663	*S. beticola*	CBS 133892	USA
KU850664	*S. beticola*	CBS 136590	New Zealand
KU850665	*S. beticola*	CBS 136699	USA
KU850666	*S. beticola*	CBS 137492	USA
KU850667	*S. beticola*	CBS 141024	Netherlands
KU850668	*S. beticola*	CBS 141025	Netherlands
KU850669	*S. beticola*	CBS 141026	Netherlands
KU850660	*S. beticola*	CBS 378.54	Canada
KU850670	*S. beticola*	GV11-196a1-3	Netherlands
KU850671	*S. beticola*	GV12-275a1	Netherlands
KU850672	*S. beticola*	GV12-276a1	Netherlands
KU850673	*S. beticola*	GV12-287a1	Netherlands
KU850674	*S. beticola*	GV12-336a1	Netherlands
KU850675	*S. beticola*	GV12-356a1	Netherlands
KU850676	*S. beticola*	GV12-367a1	Netherlands
KU850677	*S. beticola*	GV12-368a1	Netherlands
KU850678	*S. beticola*	GV12-403a1	Netherlands
KU850679	*S. beticola*	GV13-425a1	Netherlands
KU850680	*S. beticola*	GV13-436c2	Netherlands
KU850681	*S. beticola*	GV14-693a1	UK
KU850682	*S. beticola*	IFZ2013-024	Germany
KU850682	*S. beticola*	IFZ2013-035	Germany
KU850684	*S. beticola*	IFZ2014-020	Germany
KU850685	*S. botryosum*	CBS 116596	USA
AF443881	*S. botryosum*	CBS 714.68	Canada
MH206176	*S. botryosum*	M-14	China
MH206174	*S. botryosum*	M-2	China
MH206175	*S. botryosum*	M-4	China
KU850686	*S. callistephi*	CBS 527.50	USA
AY278822	*S. callistephi*	EEB 1055	Unknown
KU850782	*S. canadense*	CBS 116602	Canada
KU850783	*S. canadense*	CBS 118081	Canada
KU850781	*S. chrysanthemicola*	CBS 117255	New Zealand
KU850687	*S. drummondii*	CBS 346.83	Germany
KU850773	*S. drummondii*	CBS 716.68	USA
KU850689	*S. eturmiunum*	CBS 109845	New Zealand
KU850690	*S. eturmiunum*	CBS 122124	Greece
GQ395372	*S. eturmiunum*	CBS 122641	France
KU850692	*S. eturmiunum*	CBS 124652	China
KU850693	*S. eturmiunum*	CBS 133528	India
KU850694	*S. eturmiunum*	CBS 138495	China
KU850688	*S. eturmiunum*	CBS 668.80	Greece
KU850697	*S. gracilariae*	CBS 115179	Spain

KU850698	*S. gracilariae*	CBS 115180	Spain
KU850699	*S. gracilariae*	CBS 125060	China
KU850696	*S. gracilariae*	CBS 273.55	Unknown
KU850695	*S. gracilariae*	CBS 308.36	USA
AF443883	*S. gracilariae*	CBS 482.90	Israel
KU850700	*S. halophilum*	CBS 337.73	UK
KU850701	*S. halophilum*	CBS 410.73	UK
KU850737	*S. ixeridis*	CBS 124748	China
KU850741	*S. lancipes*	CBS 101217	New Zealand
AF443886	*S. lancipes*	CBS 116584	New Zealand
KU850742	*S. lancipes*	CBS 133314	USA
KU850743	*S. loti*	CBS 407.54	USA
KU850770	*S. lucomagnoense*	CBS 116601	Switzerland
KU850744	*S. lycii*	CBS 115192	Portugal
KU850745	*S. lycii*	CBS 116582	USA
KU850746	*S. lycii*	CBS 124982	China
KU850747	*S. lycii*	CBS 125240	China
KU850748	*S. lycii*	CBS 125241	China
AY317010	*S. lycopersici*	CBS 116585	New Caledonia
KU850753	*S. lycopersici*	CBS 116587	Dominican Republic
KU850754	*S. lycopersici*	CBS 120325	China
KU850755	*S. lycopersici*	CBS 120326	China
KU850756	*S. lycopersici*	CBS 122639	China
KU850757	*S. lycopersici*	CBS 122803	China
KU850758	*S. lycopersici*	CBS 123008	China
KU850759	*S. lycopersici*	CBS 124980	China
KU850760	*S. lycopersici*	CBS 124981	China
KU850761	*S. lycopersici*	CBS 124983	China
AY317026	*S. lycopersici*	CBS 135778	New Zealand
KU850752	*S. lycopersici*	CBS 321.87	Senegal
KU850749	*S. lycopersici*	CBS 333.73	Netherlands
KU850750	*S. lycopersici*	CBS 436.76	Indonesia
KU850751	*S. lycopersici*	CBS 463.78	Peru
MF508966	*S. lycopersici*	LJ1609270201	China
AF443891	*S. majusculum*	CBS 133424	USA
AF443891	*S. majusculum*	CBS 717.68	USA
KU850771	*S. novae-zelandiae*	CBS 138157	New Zealand
KU850772	*S. novae-zelandiae*	CBS 138295	New Zealand
KU850762	*S. paludiscirpi*	CBS 109842	USA
KU850738	*S. sarciniforme*	CBS 110049	Iran
AF443892	*S. sarciniforme*	CBS 116579	USA
KU850739	*S. sarciniforme*	CBS 116581	Iran
KU850765	*S. sarciniforme*	CBS 133723	USA
KU850740	*S. sarciniforme*	CBS 136810	Iran

KU850766	*S. sarciniforme*	CBS 138345	New Zealand
KU850763	*S. sarciniforme*	CBS 335.33	USA
KU850764	*S. sarciniforme*	CBS 364.49	USA
KU850774	*S. simmonsii*	CBS 116598	Canada
KU850775	*S. simmonsii*	CBS 116603	Canada
KU850776	*S. simmonsii*	CBS 116604	Canada
KU850777	*S. simmonsii*	CBS 133515	Canada
KU850778	*S. simmonsii*	CBS 133518	Canada
KU850779	*S. simmonsii*	CBS 133894	USA
KU850780	*S. simmonsii*	CBS 134496	Australia
KU850773	*S. simmonsii*	CBS 716.68	USA
KU850768	*S. solani*	CBS 116586	USA
KU850769	*S. solani*	CBS 118082	USA
KU850767	*S. solani*	CBS 408.54	USA
KU850784	*S. symphyti*	CBS 115268	USA
KU850785	*S. symphyti*	CBS 118796	New Zealand
KU850786	*S. symphyti*	CBS 138069	New Zealand
KU850787	*S. symphyti*	CBS 138070	New Zealand
KU850788	*S. trifolii*	CBS 116580	USA
KU850789	*S. triglochinicola*	CBS 718.68	UK
KU850718	*S. vesicarium*	CBS 109843	New Zealand
KU850719	*S. vesicarium*	CBS 109844	USA
KU850720	*S. vesicarium*	CBS 115182	South Africa
KU850721	*S. vesicarium*	CBS 115204	Portugal
KU850722	*S. vesicarium*	CBS 122640	China
KU850723	*S. vesicarium*	CBS 123005	China
KU850724	*S. vesicarium*	CBS 123803	China
KU850725	*S. vesicarium*	CBS 124279	Denmark
KU850726	*S. vesicarium*	CBS 124747	China
KU850727	*S. vesicarium*	CBS 124749	China
KU850728	*S. vesicarium*	CBS 124751	China
KU850729	*S. vesicarium*	CBS 124752	China
KU850730	*S. vesicarium*	CBS 125242	China
KU850731	*S. vesicarium*	CBS 133474	USA
KU850732	*S. vesicarium*	CBS 133737	Australia
KU850733	*S. vesicarium*	CBS 133905	USA
KU850734	*S. vesicarium*	CBS 133914	USA
KU850735	*S. vesicarium*	CBS 138138	Netherlands
KU850702	*S. vesicarium*	CBS 155.24	Unknown
KU850708	*S. vesicarium*	CBS 156.45	Netherlands
KU850703	*S. vesicarium*	CBS 157.24	Unknown
KU850704	*S. vesicarium*	CBS 184.25	UK
AF443884	*S. vesicarium*	CBS 191.86	India
KU850715	*S. vesicarium*	CBS 192.86	Australia

KU850714	*S. vesicarium*	CBS 205.82	Netherlands
KU850705	*S. vesicarium*	CBS 273.31	Unknown
KU850706	*S. vesicarium*	CBS 274.31	Unknown
KU850707	*S. vesicarium*	CBS 307.36	Tunisia
KU850716	*S. vesicarium*	CBS 311.92	Netherlands
KU850709	*S. vesicarium*	CBS 322.49	Netherlands
KU850711	*S. vesicarium*	CBS 368.59	Denmark
KU850710	*S. vesicarium*	CBS 370.51	Netherlands
KU850713	*S. vesicarium*	CBS 406.76	Germany
KU850717	*S. vesicarium*	CBS 486.92	Netherlands
KU850712	*S. vesicarium*	CBS 715.68	Canada
KU850736	*S. vesicarium*	GV11-355a1-2	Netherlands
MH206177	*S. vesicarium*	M-6	China
***calmodulin* gene**			
KU850791	*S. amaranthi*	CBS 124650	China
KU850792	*S. amaranthi*	CBS 124651	China
KU850793	*S. amaranthi*	CBS 124746	China
KU850794	*S. amaranthi*	CBS 124750	Chile
KU850795	*S. amaranthi*	CBS 124753	China
KU850796	*S. amaranthi*	CBS 124984	China
KU850797	*S. amaranthi*	CBS 124985	China
KU850798	*S. amaranthi*	CBS 124989	New Zealand
KU850798	*S. amaranthi*	CBS 136589	New Zealand
KU850799	*S. armeriae*	CBS 338.73	UK
KU850800	*S. astragali*	CBS 116583	Japan
KU850802	*S. beticola*	CBS 116599	Canada
KU850803	*S. beticola*	CBS 133512	Canada
KU850804	*S. beticola*	CBS 133892	USA
KU850805	*S. beticola*	CBS 136590	New Zealand
KU850806	*S. beticola*	CBS 136699	USA
KU850807	*S. beticola*	CBS 137492	USA
KU850808	*S. beticola*	CBS 141024	Netherlands
KU850809	*S. beticola*	CBS 141025	Netherlands
KU850810	*S. beticola*	CBS 141026	Netherlands
KU850801	*S. beticola*	CBS 378.54	Canada
KU850811	*S. beticola*	GV11-196a1-3	Netherlands
KU850812	*S. beticola*	GV12-275a1	Netherlands
KU850813	*S. beticola*	GV12-276a1	Netherlands
KU850814	*S. beticola*	GV12-287a1	Netherlands
KU850815	*S. beticola*	GV12-336a1	Netherlands
KU850816	*S. beticola*	GV12-356a1	Netherlands
KU850817	*S. beticola*	GV12-367a1	Netherlands
KU850818	*S. beticola*	GV12-368a1	Netherlands
KU850819	*S. beticola*	GV12-403a1	Netherlands

KU850820	*S. beticola*	GV13-425a1	Netherlands
KU850821	*S. beticola*	GV13-436c2	Netherlands
KU850822	*S. beticola*	GV14-693a1	UK
KU850823	*S. beticola*	IFZ2013-024	Germany
KU850823	*S. beticola*	IFZ2013-035	Germany
KU850825	*S. beticola*	IFZ2014-020	Germany
KU850827	*S. botryosum*	CBS 116596	USA
KU850826	*S. botryosum*	CBS 714.68	Canada
MH206180	*S. botryosum*	M-14	China
MH206178	*S. botryosum*	M-2	China
MH206179	*S. botryosum*	M-4	China
KU850828	*S. callistephi*	CBS 527.50	USA
JQ646103	*S. callistephi*	EEB 1055	Unknown
KU850932	*S. canadense*	CBS 116602	Canada
KU850933	*S. canadense*	CBS 118081	Canada
KU850931	*S. chrysanthemicola*	CBS 117255	New Zealand
KU850829	*S. drummondii*	CBS 346.83	Germany
KU850923	*S. drummondii*	CBS 716.68	USA
KU850831	*S. eturmiunum*	CBS 109845	New Zealand
KU850832	*S. eturmiunum*	CBS 122124	Greece
KU850833	*S. eturmiunum*	CBS 122641	France
KU850834	*S. eturmiunum*	CBS 124652	China
KU850835	*S. eturmiunum*	CBS 133528	India
KU850836	*S. eturmiunum*	CBS 138495	China
KU850830	*S. eturmiunum*	CBS 668.80	Greece
KU850840	*S. gracilariae*	CBS 115179	Spain
KU850841	*S. gracilariae*	CBS 115180	Spain
KU850842	*S. gracilariae*	CBS 125060	China
KU850838	*S. gracilariae*	CBS 273.55	Unknown
KU850837	*S. gracilariae*	CBS 308.36	USA
KU850839	*S. gracilariae*	CBS 482.90	Israel
KU850843	*S. halophilum*	CBS 337.73	UK
KU850844	*S. halophilum*	CBS 410.73	UK
KU850881	*S. ixeridis*	CBS 124748	China
KU850885	*S. lancipes*	CBS 101217	New Zealand
KU850886	*S. lancipes*	CBS 116584	New Zealand
KU850887	*S. lancipes*	CBS 133314	USA
KU850888	*S. loti*	CBS 407.54	USA
KU850920	*S. lucomagnoense*	CBS 116601	Switzerland
KU850889	*S. lycii*	CBS 115192	Portugal
KU850890	*S. lycii*	CBS 116582	USA
KU850891	*S. lycii*	CBS 124982	China
KU850892	*S. lycii*	CBS 125240	China
KU850893	*S. lycii*	CBS 125241	China

KU850898	*S. lycopersici*	CBS 116585	New Caledonia
KU850899	*S. lycopersici*	CBS 116587	Dominican Republic
KU850900	*S. lycopersici*	CBS 120325	China
KU850901	*S. lycopersici*	CBS 120326	China
KU850902	*S. lycopersici*	CBS 122639	China
KU850903	*S. lycopersici*	CBS 122803	China
KU850904	*S. lycopersici*	CBS 123008	China
KU850905	*S. lycopersici*	CBS 124980	China
KU850906	*S. lycopersici*	CBS 124981	China
KU850907	*S. lycopersici*	CBS 124983	China
KU850908	*S. lycopersici*	CBS 135778	New Zealand
KU850897	*S. lycopersici*	CBS 321.87	Senegal
KU850894	*S. lycopersici*	CBS 333.73	Netherlands
KU850895	*S. lycopersici*	CBS 436.76	Indonesia
KU850896	*S. lycopersici*	CBS 463.78	Peru
MG742412	*S. lycopersici*	LJ1609270201	China
KU850910	*S. majusculum*	CBS 133424	USA
KU850909	*S. majusculum*	CBS 717.68	USA
KU850921	*S. novae-zelandiae*	CBS 138157	New Zealand
KU850922	*S. novae-zelandiae*	CBS 138295	New Zealand
KU850911	*S. paludiscirpi*	CBS 109842	USA
KU850882	*S. sarciniforme*	CBS 110049	Iran
KU850914	*S. sarciniforme*	CBS 116579	USA
KU850883	*S. sarciniforme*	CBS 116581	Iran
KU850915	*S. sarciniforme*	CBS 133723	USA
KU850884	*S. sarciniforme*	CBS 136810	Iran
KU850916	*S. sarciniforme*	CBS 138345	New Zealand
KU850912	*S. sarciniforme*	CBS 335.33	USA
KU850913	*S. sarciniforme*	CBS 364.49	USA
KU850924	*S. simmonsii*	CBS 116598	Canada
KU850925	*S. simmonsii*	CBS 116603	Canada
KU850926	*S. simmonsii*	CBS 116604	Canada
KU850927	*S. simmonsii*	CBS 133515	Canada
KU850928	*S. simmonsii*	CBS 133518	Canada
KU850929	*S. simmonsii*	CBS 133894	USA
KU850930	*S. simmonsii*	CBS 134496	Australia
KU850923	*S. simmonsii*	CBS 716.68	USA
KU850918	*S. solani*	CBS 116586	USA
KU850919	*S. solani*	CBS 118082	USA
KU850917	*S. solani*	CBS 408.54	USA
KU850934	*S. symphyti*	CBS 115268	USA
KU850935	*S. symphyti*	CBS 118796	New Zealand
KU850936	*S. symphyti*	CBS 138069	New Zealand
KU850937	*S. symphyti*	CBS 138070	New Zealand

KU850938	*S. trifolii*	CBS 116580	USA
KU850939	*S. triglochinicola*	CBS 718.68	UK
KU850862	*S. vesicarium*	CBS 109843	New Zealand
KU850863	*S. vesicarium*	CBS 109844	USA
KU850864	*S. vesicarium*	CBS 115182	South Africa
KU850865	*S. vesicarium*	CBS 115204	Portugal
KU850866	*S. vesicarium*	CBS 122640	China
KU850867	*S. vesicarium*	CBS 123005	China
KU850868	*S. vesicarium*	CBS 123803	China
KU850869	*S. vesicarium*	CBS 124279	Denmark
KU850870	*S. vesicarium*	CBS 124747	China
KU850871	*S. vesicarium*	CBS 124749	China
KU850872	*S. vesicarium*	CBS 124751	China
KU850873	*S. vesicarium*	CBS 124752	China
KU850874	*S. vesicarium*	CBS 125242	China
KU850875	*S. vesicarium*	CBS 133474	USA
KU850876	*S. vesicarium*	CBS 133737	Australia
KU850877	*S. vesicarium*	CBS 133905	USA
KU850878	*S. vesicarium*	CBS 133914	USA
KU850879	*S. vesicarium*	CBS 138138	Netherlands
KU850845	*S. vesicarium*	CBS 155.24	Unknown
KU850851	*S. vesicarium*	CBS 156.45	Netherlands
KU850846	*S. vesicarium*	CBS 157.24	Unknown
KU850847	*S. vesicarium*	CBS 184.25	UK
KU850858	*S. vesicarium*	CBS 191.86	India
KU850859	*S. vesicarium*	CBS 192.86	Australia
KU850857	*S. vesicarium*	CBS 205.82	Netherlands
KU850848	*S. vesicarium*	CBS 273.31	Unknown
KU850849	*S. vesicarium*	CBS 274.31	Unknown
KU850850	*S. vesicarium*	CBS 307.36	Tunisia
KU850860	*S. vesicarium*	CBS 311.92	Netherlands
KU850852	*S. vesicarium*	CBS 322.49	Netherlands
KU850854	*S. vesicarium*	CBS 368.59	Denmark
KU850853	*S. vesicarium*	CBS 370.51	Netherlands
KU850856	*S. vesicarium*	CBS 406.76	Germany
KU850861	*S. vesicarium*	CBS 486.92	Netherlands
KU850855	*S. vesicarium*	CBS 715.68	Canada
KU850880	*S. vesicarium*	GV11-355a1-2	Netherlands
MH206181	*S. vesicarium*	M-6	China
28S rRNA			
KX228349	*S. beticola*	-	Netherlands
MH878201	*S. beticola*	-	Netherlands
KC584345	*S. botryosum*	CBS 714.68	Netherland
MH870931	*S. botryosum*	CBS 714.68	Netherland

MH868266	*S. callistephi*	CBS 527.50	USA
MH874431	*S. eturmiunum*	CBS 109845	New Zealand
MK431468	*S. eturmiunum*	JAC12722	New Zealand
AY849955	*S. halophilum*	CBS 410.73	UK
MH872400	*S. halophilum*	CBS 337.73	UK
MH868917	*S. loti*	CBS 407.54	USA
MH874554	*S. lycopersici*	CBS 116585	Netherland
MH874764	*S. lycopersici*	CBS 122803	Netherland
MH870932	*S. majusculum*	CBS 717.68	USA
MH874428	*S. paludiscirpi*	CBS 109842	USA
MH868918	*S. solani*	CBS 408.54	Netherland
MH870933	*S. triglochinicola*	CBS 718.68	UK
MH255549	*S. vesicarium*	Am95	Iran
MH873624	*S. vesicarium*	CBS 191.86	India
MH873625	*S. vesicarium*	CBS 192.86	Australia
MH874429	*S. vesicarium*	CBS 109843	New Zealand
MH874430	*S. vesicarium*	CBS 109844	USA
MH874919	*S. vesicarium*	CBS 124749	China
ATPase			
AB828244	*S. lycopersici*	MAFF 731071	Japan
AY329302	*P. paludiscirpi*	EGS 31-016	Unknown
AY329271	*S. botryosum*	EGS08-069	Canada
AY329321	*S. callistephi*	NO 0536	USA
AY329301	*S. eturmiunum*	EGS29-099	New Zealand
KF479196	*S. globuliferum*	SWp202	Unknown
AY329291	*S. gracilariae*	EGS 37-073	Israel
AY329283	*S. lancipes*	EGS46-182	New Zealand
AY329322	*S. loti*	NO 0770	USA
AY329323	*S. loti*	NO 1364	USA
AB828245	*S. lycopersici*	MAFF 744030	Japan
AB828246	*S. lycopersici*	MAFF 712285	Japan
AB828247	*S. lycopersici*	MAFF 235583	Japan
AB828248	*S. lycopersici*	KuNBY1	Japan
AB828249	*S. lycopersici*	KuNBY2	Japan
AB828250	*S. lycopersici*	KuNBY3	Japan
AB828251	*S. lycopersici*	KuNBY4	Japan
AB979970	*S. lycopersici*	MAFF 238342	Japan
AB979971	*S. lycopersici*	MAFF 238863	Japan
AB979972	*S. lycopersici*	MAFF 243758	Japan
AB979973	*S. lycopersici*	MAFF 306242	Japan
AB979974	*S. lycopersici*	MAFF 306243	Japan
AB979975	*S. lycopersici*	MAFF 306244	Japan
AB979976	*S. lycopersici*	MAFF 306245	Japan
AB979977	*S. lycopersici*	MAFF 306719	Japan

AB979978	*S. lycopersici*	MAFF 425273	Japan
KC160512	*S. lycopersici*	NAAS12164	Korea
KC160513	*S. lycopersici*	NAAS12167	Korea
KC160514	*S. lycopersici*	NAAS12168	Korea
KF483129	*S. lycopersici*	SSN-T03	Malaysia
KF483130	*S. lycopersici*	SSN-E12	Malaysia
KF483132	*S. lycopersici*	SSN-L05	Malaysia
KU599921	*S. lycopersici*	NC15-378	Korea
LC413542	*S. lycopersici*	CAR8	Japan
MF357690	*S. lycopersici*	Unknown	China
AY329299	*S. majusculum*	EGS16-068	USA
AB979979	*S. sarciniforme*	MAFF 305456	Japan
AY329289	*S. sarciniforme*	EGS38-121	USA
AY329295	*S. sarciniforme*	EGS29-188	Iran
AY329292	*S. trifolii*	EGS12-142	USA
AY329277	*S. triglochinicola*	EGS36-118	UK
AY329272	*S. vesicarium*	EGS36-138	India
AY329274	*S. vesicarium*	EGS36-088	Australia
AY329300	*S. vesicarium*	EGS29-089	USA
AY329303	*S. vesicarium*	EGS48-095	New Zealand
LC413540	*S. vesicarium*	CAR4	China
LC413541	*S. vesicarium*	CAR6	China
EF-1 alpha			
AY324769	*P. paludiscirpi*	EGS 31-016	
AY324750	*S. callistephi*	NO 0536	USA
AY324709	*S. gracilariae*	EGS 37-073	Israel
AY324742	*S. lancipes*	EGS46-182	New Zealand
AY324775	*S. loti*	NO 0770	USA
AY324776	*S. loti*	NO 1364	USA
AB828253	*S. lycopersici*	KuNBY4	Japan
AB828252	*S. lycopersici*	MAFF 731071	Japan
AB828254	*S. lycopersici*	MAFF 744030	Japan
AB828255	*S. lycopersici*	MAFF 235583	Japan
AB828256	*S. lycopersici*	MAFF 712285	Japan
AB828257	*S. lycopersici*	KuNBY1	Japan
AB828258	*S. lycopersici*	KuNBY2	Japan
AB828259	*S. lycopersici*	KuNBY3	Japan
AB979910	*S. lycopersici*	MAFF 238342	Japan
AB979911	*S. lycopersici*	MAFF 238863	Japan
AB979912	*S. lycopersici*	MAFF 243758	Japan
AB979913	*S. lycopersici*	MAFF 306242	Japan
AB979914	*S. lycopersici*	MAFF 306243	Japan
AB979915	*S. lycopersici*	MAFF 306244	Japan
AB979916	*S. lycopersici*	MAFF 306245	Japan

AB979917	*S. lycopersici*	MAFF 306719	Japan
AB979918	*S. lycopersici*	MAFF 425273	Japan
AY324761	*S. lycopersici*	EGS46-001	Canada
KR911804	*S. lycopersici*	01	China
KR911805	*S. lycopersici*	02	China
KR911806	*S. lycopersici*	03	China
KR911807	*S. lycopersici*	04	China
LC413539	*S. lycopersici*	CAR8	Japan
AY324710	*S. majusculum*	EGS16-068	USA
AB979919	*S. sarciniforme*	MAFF 305456	Japan
AY324743	*S. sarciniforme*	EGS38-121	USA
AY324746	*S. sarciniforme*	EGS29-188	Iran
AY324759	*S. solani*	EGS41-135	Canada
AY324760	*S. solani*	EGS42-027	Canada
AY324744	*S. trifolii*	EGS12-142	USA
AY324753	*S. triglochinicola*	EGS36-118	UK
AY324675	*S. vesicarium*	EGS36-138	India
AY324676	*S. vesicarium*	EGS36-088	Australia
AY324711	*S. vesicarium*	EGS29-089	USA
AY324713	*S. vesicarium*	EGS48-095	New Zealand
LC413537	*S. vesicarium*	CAR4	China
LC413538	*S. vesicarium*	CAR6	China
MH628121	*S. vesicarium*	OA20	Canada
MH628122	*S. vesicarium*	OA27	Canada
MH628123	*S. vesicarium*	OO31	Canada
MH628124	*S. vesicarium*	NO35	Canada
MH628125	*S. vesicarium*	NO36	Canada
MH628126	*S. vesicarium*	OA46	Canada
MH628127	*S. vesicarium*	OA48	Canada
MH628128	*S. vesicarium*	NA51	Canada
histidine kinase			
EU711363	*S. vesicarium*	Sv173'04	Italy
EU711364	*S. vesicarium*	Sv346	Italy
EU711365	*S. vesicarium*	Sv448	Italy
EU711366	*S. vesicarium*	Sv524	Italy
EU711367	*S. vesicarium*	Sv464	Italy
EU711368	*S. vesicarium*	Sv222'0	Italy
EU711369	*S. vesicarium*	Sv479	Italy
EU711370	*S. vesicarium*	Sv439	Italy
EU711371	*S. vesicarium*	Sv563	Italy
